# Prevalence, characteristics, and psychological outcomes of workplace cyberbullying during the COVID-19 pandemic in Japan: a cross-sectional online survey

**DOI:** 10.1186/s12889-022-13481-6

**Published:** 2022-05-31

**Authors:** Tomohiko Ikeda, Daisuke Hori, Hiroaki Sasaki, Yu Komase, Shotaro Doki, Tsukasa Takahashi, Yuichi Oi, Yu Ikeda, Yo Arai, Kei Muroi, Mami Ishitsuka, Asako Matsuura, Wyi Go, Ichiyo Matsuzaki, Shinichiro Sasahara

**Affiliations:** 1 Graduate School of Comprehensive Human Sciences, University of Tsukuba, 1-1-1 Tennodai, Tsukuba, Ibaraki 305-8575 Japan; 2 Faculty of Medicine, University of Tsukuba, 1-1-1 Tennodai, Tsukuba, Ibaraki 305-8575 Japan; 3 International Institute for Integrative Sleep Medicine, University of Tsukuba, 1-1-1 Tennodai, Tsukuba, Ibaraki 305-8575 Japan; 4 Department of Mental Health, Graduate School of Medicine, The University of Tokyo, Japan. 7-3-1 Hongo, Bunkyo-ku, Tokyo, 113-0033 Japan; 5 Japan Society for the Promotion of Science, 5-3-1 Kojimachi, Chiyoda-ku, Tokyo, 102-0083 Japan

**Keywords:** Cyberbullying, Internet use, Japan, Teleworking, Worker, Workplace bullying

## Abstract

**Background:**

The rapid introduction of teleworking due to the coronavirus disease 2019 pandemic has led to concerns about increases in cyberbullying (CB) worldwide. However, little is known about workplace CB in non-Western countries. The first objective was to clarify the prevalence and characteristics regarding workplace CB victimization in Japan. The second objective was to demonstrate the psychological outcomes of CB victimization in combination with traditional bullying (TB).

**Methods:**

We conducted an anonymous, cross-sectional, Internet-based survey targeting regular employees in Japan (*N* = 1200) in January 2021. We investigated CB victimization using the Inventory of Cyberbullying Acts at Work and TB victimization by using the Short Negative Act Questionnaire. Possible explanatory factors for TB/CB victimization were sociodemographic variables, personality trait, chronic occupational stress, organizational climate, and gratitude at work. We also measured psychological distress, insomnia, and loneliness to assess adverse effects of workplace bullying. Two-step cluster analysis was used in determining the patterns combined with TB and CB victimization. Hierarchical binomial logistic regression analysis was used.

**Results:**

In total, 8.0% of employees reported experiencing CB on a weekly basis. CB victimization was associated with younger age, managerial position, higher qualitative workload, and active information dissemination via the Internet, and frequency of teleworking. Three clusters based on TB and CB victimization patterns were identified: those who belong to the first cluster suffered neither from TB and CB (81.0%), the second cluster suffered only from TB (14.3%), and the third cluster suffered from both TB and CB (4.8%). The third cluster exhibited higher odds ratios (ORs) and 95% confidence intervals (CIs) for psychological distress (OR = 12.63, 95% CI = 4.20–38.03), insomnia (OR = 6.26, 95% CI = 2.80–14.01), and loneliness (OR = 3.24, 95% CI = 1.74–6.04) compared to the first cluster.

**Conclusions:**

These findings firstly clarify the prevalence and correlated factors of CB victimization among employees in Japan. Further, we showed that psychological wellbeing can be impaired by the coexistence of TB and CB. Our research could be the first step to develop the effective countermeasures against workplace CB.

**Supplementary Information:**

The online version contains supplementary material available at 10.1186/s12889-022-13481-6.

## Background

Workplace bullying has become a serious threat for employees’ social, physical, and psychological well-being [1–3]. Workplace bullying refers to a situation in which a person is repeatedly subjected to harassment, abuse, or social exclusion in his/her workplace over a period of time and finds it difficult to defend him/herself against these maltreatments [4, 5]. The development of information and communication technology (ICT) devices and the spread of the coronavirus disease 2019 (COVID-19) pandemic have accelerated the trend of teleworking [6, 7]. The COVID-19 pandemic has a huge impact on people’s mental health all across the world [8–10]. In addition, it is feared that workplace cyberbullying (CB), which involves electronic forms of contact for bullying, will increase as more people begin working remotely [11–13]. In this manuscript, bullying victimization in which electronic forms of contact are not specifically involved is defined as traditional bullying (TB). Exposure to workplace bullying can shatter victims’ basic assumption of self-worth and increase negative views of themselves, others, and the world [14]. Victims of CB report higher levels of turnover intention and anxiety, less optimism, worse performance, and reduced psychological and physical well-being [5, 11, 15–17].

CB is relatively a new phenomenon that shares the same nature as TB. It has been pointed out that CB can occur as an extension of TB [18]. However, CB has three distinguishing characteristics that are not found in TB. The first one is anonymity. Sometimes the victim of CB does not know who the perpetrator is. This anonymity allows the perpetrator of CB to escape social constraints and responsibilities [19, 20]. The second is dissemination. For example, if abuse or slander is leaked through videos or text messages, it can be exposed to a very large number of people [21]. The third is that it is location- and time-independent; CB can occur anywhere and anytime throughout the day [22]. Research on CB has primarily emerged as a problem to solve for adolescence; research on CB among employees remains limited [23].

A meta-analysis demonstrated that a considerable number of employees are being victimized by workplace bullying [24]. Precedent studies have reported that CB victimization rates differ greatly by sample populations, reporting periods (6–12 months), and measurement scales [18, 25, 26]. For example, in a survey of Australian manufacturing workers, 10.7% of respondents were cyberbullied on at least a weekly basis in the last 6 months [27]. A survey of British trainee doctors showed that 46.2% had experienced some form of CB in the past 6 months [28]. In a study of New Zealand workers, 2.8% participants experienced at least two forms of CB at least weekly for the last 6 months [15]. Although few studies of CB at work have been reported from Asian countries, Park and Choi [29] reported that the prevalence of CB victimization among nurses in Korea was 8.0%.

Understanding the factors underlying CB is important to develop effective countermeasures. Regarding individual factors, a presence of CB victimization in the workplace has been shown to be associated with male sex, managerial position, poor physical health, and lower levels of optimism [15, 16]. Some studies have also reported that certain professions, such as politicians, are at a higher risk for CB victimization [30]. CB victimization is sometimes recognized by those who enjoy online activities, such as spending time on social networking sites (SNSs) [31, 32]. Workers with higher levels of neuroticism and conscientiousness and low levels of agreeableness are more likely to report TB victimization [33]. Although no relationship between CB victimization and personality traits has been reported among workers, studies of young adults have shown that extroversion and openness are predictors of CB victimization [34]. Regarding work-related factors, precedent studies have reported a significant association between low support from managers or colleagues and CB victimization [15, 35]. In addition, a poor organizational climate [36] described as “distrustful and suspicious” and “rigid and rule-based”, has been shown to be associated with CB behavior [35]. In the current study, we also tested the effect of gratitude at work, defined as the tendency to recognize and appreciate the impact of various aspects of work on one’s life [37, 38], on workplace bullying. Although precedent research has proposed that gratitude is a promising resource to protect adolescents from adverse outcomes caused by CB victimization [39], it was not examined in working population.

As mentioned above, research on CB in the workplace has been accumulating in the last decade. The main limitation of precedent research is that most studies have been conducted in Western countries, even though TB/CB in the working population is perceived as a serious problem in other cultural contexts, such as in Japan. The Japanese work environment is based on simultaneous recruiting of new graduates and lifetime employment [40], and mid-career hiring and job change opportunities are still developing. The Japanese government cites examples of workplace bullying such as hitting things, yelling, verbal abuse, invasion of privacy, neglect, and not giving work [41]. They mentioned that it is difficult to draw a line between workplace bullying and work-related guidance, especially when it was given by a supervisor to a subordinate. In the government survey, the one third of victims said they took no actions after being harassed in the workplace, because they thought “it would not solve anything” or “it would be detrimental for my job” [42]. This situation carries a risk that workplace bullying could persist. Regarding CB, a young woman’s suicide due to slander on SNSs, who was on a popular reality TV show, sparked national debate in 2020 [43]. The number of consultations received by the Illegal Harmful Hotline, a government-supported consultation service for illegal and harmful information on the Internet, has increased from 1,337 in fiscal year (FY) 2010 to 5,198 in FY 2019 [44]. However, despite its widespread recognition, no survey has revealed the prevalence of CB using an internationally used assessment tool, its antecedents, or its negative health outcomes in Japan. The percentage of employees who engage in teleworking doubled from 9.8% in FY 2019 to 19.7% in FY 2020 [45]. Considering that teleworking is becoming increasingly common and has been accelerated by the COVID-19 pandemic, more information on CB in the workplace is needed to fill the knowledge gap and develop effective countermeasures.

Our research questions are the following: What percentage of workers were suffering from CB? What kinds of factors are associated with CB victimization? Is CB victimization associated with frequencies of teleworking? Is CB victimization associated with psychological well-being? To our knowledge, no studies have attempted to answer these questions in Japan. To address these issues, we conducted an anonymous, cross-sectional, Internet-based survey targeting nationwide workers in Japan. At that time, the Tokyo metropolitan area was under its second emergency declaration due to the spread of COVID-19 (from January 8 to March 21, 2021).

Our analysis is divided into two parts. First, we define participants as having been victimized if they experienced any kind of event suggested by the questionnaire at least weekly during the last 6 months [4] and examine the following hypothesis. Although our primary focus was on CB, we also examined TB to gain a better understanding of the characteristics of CB.

### Hypothesis 1

Factors such as sociodemographic characteristics, personality traits, information dissemination on SNSs, and work circumstances make people vulnerable to CB victimization.

In the second part of analysis, we focus on the negative consequences of CB on employees’ psychological well-being. The limitations of the majority of the precedent studies are that they did not consider the consequences of the combination of TB and CB. Because TB and CB have been shown to coexist, we considered it more appropriate to treat them as mutually dependent variables. We used a two-step cluster analysis to identify groups of respondents with similar patterns of TCB (traditional and cyber bullying) victimization. We expected that it would reveal distinctive patterns of TCB victimization, as precedent studies have shown in a latent class cluster model [46, 47]. Therefore, our second hypothesis is the following.

### Hypothesis 2

The co-existence of TB and CB victimization demonstrate severe effects on psychological well-being (e.g., psychological distress, insomnia, and loneliness).

The present study aims to replicate and add to the existing literature in the context of the Japanese workforce. Workplace bullying is increasingly recognized as a public health concern for policymakers and stakeholders. Since the use of the ICT in the workplace is expected to expand during the COVID-19 pandemic, this study could be the first step in clarifying the actual condition of CB victimization among employees, which would also be helpful for the early detection and prevention of the negative outcomes of workplace bullying victimization.

## Methods

### Sampling and data collection

An anonymous, cross-sectional, self-administered Internet-based survey targeting regular employees aged 20–64 years who were not on leave was conducted in January 2021. All participants were registered monitors of an Internet research company. Executives and self-employed workers were not targeted. The survey was limited to regular employees because the majority of teleworkers were in regular employment at the time [48]. On the basis of hypothesis 1, G*Power 3.1.9.7 was used to calculate the sample size [49]. We assumed that 30% of the employees were teleworkers at the time and approximated a binomial distribution (teleworkers vs. non-teleworkers). The overall prevalence of CB victimization was assumed to be about 10%, while that among teleworkers was assumed to be about 16%. The α value was set at 0.05 and the power (= 1 – β) at 0.80. As a result, a total sample size of 1,152 was needed. Therefore, completed questionnaires were collected until valid responses had been received from 1,200 employees. The ratio of male to female workers was set as 2:1 (800:400), in line with the ratio of male to female regular employees nationwide [50].

The survey was entitled “Survey on positive and negative aspects of work” and excluded the term “bullying” throughout the questionnaire to minimize the preconceptions of the respondents. All participants were given a URL code they could use to access the survey form. The purpose of the survey, the voluntary nature of participation, the anonymity and confidentiality of the responses, a guarantee of secure data management, and the intent to publish the results were clearly explained to all participants. All workers were required to read and agree to the online consent form before responding to the questionnaire. Those who answered in an extremely short time or who gave all answers in the same row were considered to provide invalid responses and therefore not included.

### Questionnaire components

#### S-NAQ and ICA-W

In epidemiological studies, workplace bullying victimization is typically assessed by a two-types of self-administered questionnaire. One is to give the definition of workplace bullying and then ask if the person has had such an experience [51]. The other is to measure the frequency at which respondents have been subjected to various types of negative contacts (e.g. “Being ignored or excluded”, “Persistent criticism of your work and effort”), using a scale such as the Negative Act Questionnaire (NAQ) [46]. Since both methods are based on self-administered questionnaires, they depend to a considerable extent on the subjective criteria of the victims. However, the latter require less for the respondents in processing the information cognitively and emotionally. In this study, we adopted the latter and used the short form of NAQ (S-NAQ) [47] to assess TB victimization and used the Inventory of Cyberbullying Acts at Work (ICA-W) [25] to assess CB victimization.

The S-NAQ is composed of nine items extracted from the 22 items on the NAQ-revised. The Japanese version of the NAQ-revised was developed in a precedent study [52]. The participants were asked to select the frequency with which they had been exposed to the nine patterns of negative behaviors in the past 6 months, from “never”, “sometimes (once every 2 or 3 months)”, “about once a month”, “about once a week”, and “every day”. The ICA-W Japanese version is composed of 10 items [25, 53]. Although various questionnaires have been developed for assessing CB victimization, the ICA-W has a relatively small number of questions [54]. In this study, we used the ICA-W to reduce the burden on the respondents. The participants were asked to select the frequency with which they had been exposed to 10 patterns of negative behaviors via ICT in the past 6 months, from “never”, “once”, “every month”, “every week”, and “every day”.

#### Possible explanatory factors for TB/CB victimization

We used the following items as possible explanatory factors for TB/CB victimization: age, sex, marital status, annual household income, educational attainment, residential area, type of industry, type of work, number of employees in the workplace, average working hours per week, and frequency of teleworking. Regarding the level of information dissemination via internet, we created a questionnaire based on a survey conducted by the Japanese Ministry of Internal Affairs and Communications on the use of Internet media [55]. Categories where the total number of respondents was less than 3% (36 respondents) were integrated into the “Others” category. See Additional file 1 for detailed answer options and categories. To measure frequency of teleworking, we asked the participants to respond to the question “How often do you currently telework?” using the following four options: “almost never”, “about 1 to 3 times a month”, “about 1 to 3 times a week”, and “almost every day”, with reference to a survey conducted by the Tokyo Metropolitan Government [56].

We also used the following scales as possible explanatory factors for TB/CB victimization: Japanese version of the Ten-Item Personality Inventory (TIPI-J), Brief Scales for Job Stress (BSJS), the 12-item Organizational Climate Scale (OCS-12), and the Gratitude at Work Scale (GAWS).

The TIPI-J is composed of 10 items with 7 Likert scales to assess the five major personality traits [57, 58]: extraversion, agreeableness, conscientiousness, neuroticism, and openness. The TIPI-J has been shown to have sufficient reliability and validity [58]. The five personality traits and their associated characteristics are as follows [34]: extraversion: sociable, energetic, and talkative; agreeableness: warmth, cooperativeness, and helpfulness; conscientiousness: discipline, predictability, and orderliness; neuroticism: moodiness, anxiety, and emotional instability; and openness: creativity, intellectualism, and a preference for novelty.

We used the BSJS [59], which was developed as an assessment tool for job stress in reference to the Job Demand–Control–Support (JDCS) model [60], similar to the Job Content Questionnaire [61]. The BSJS is composed of 20 items with 4 Likert scales, such as “I have too much work to do”. These items are classified into six subscales. In the present study, we used four subscales—“quantitative workload”, “qualitative workload”, “job control”, and “support from colleagues and superiors”—in accordance with the JDCS model [60]. The first two categories are stress enhancing factors, and the last two are stress buffering factors. The BSJS has been shown to have sufficient reliability and validity [59].

We used the OCS-12 [62], a scale developed in Japan, as an assessment instrument for organizational climate. The OCS-12 is a self-administered questionnaire consisting of 12 items with 2 Likert scales categorized into two subscales—the “tradition scale” and the “organizational environment scale”—with six items each. Higher tradition scale scores indicate a coercive, commanding, and legalistic organizational climate, whereas higher organizational environmental scale scores indicate an organizational climate with high levels of employee participation and rational organizational management.

We used the GAWS [37] as an assessment tool for workplace-specific gratitude. The GAWS has been translated into Japanese and has shown sufficient validity and reliability [63]. The GAWS is a self-administered questionnaire composed of 10 items with 5 Likert scales classified into the following two subscales: “gratitude for supportive work environment” and “gratitude for meaningful work”.

#### Psychological outcomes for TCB-victimization clusters

We used the following items to reveal the psychological outcomes of TB/CB victimization clusters: the Japanese version of the 6-item Kessler Psychological Distress Scale (K6), the Athens Insomnia Scale (AIS), and the Japanese version of the Three-Item Loneliness Scale (TIL-J).

The K6 is a widely used international scale for measuring psychological distress [64]. The validity and reliability of the Japanese version of the K6 have been confirmed [65]. The respondents were asked to select one of the following options regarding the frequency of feelings of “nervousness”, “hopelessness”, “discomfort”, “feeling depressed”, “not being able to do anything”, and “thinking I am worthless” in the past 30 days: “never”, “a little”, “sometimes”, “most of the time”, and “always”. A higher total score (range, 0–24) indicates a higher level of depression, and a total score ≥ 5 indicates psychological distress. The AIS is a self-administered questionnaire that assesses insomnia based on the 10th revision of the International Classification of Diseases criteria [66]. The Japanese version of the AIS has been validated [67]. The total score ranges from 0 to 24. The respondents were then categorized into two groups: an insomnia group (AIS total score ≥ 6) and a no insomnia group (AIS total score ≤ 5). Loneliness is defined as a subjective negative feeling arising from the perception of a discrepancy between one’s actual and ideal states of interpersonal relationships [68]. The TIL-J is as an assessment tool for loneliness that has been shown to have sufficient reliability and validity [69, 70], and is composed of three items. The TIL-J score was calculated by adding the scores for the three items. A higher TIL-J score indicates higher loneliness. We dichotomized a loneliness group (total score of 3–5) and a no loneliness group (total score of 6–9) based on the cutoff reported in a precedent study [71].

### Statistical analysis

#### Investigating possible explanatory factors for TB/CB victimization

We conducted factor analysis on the S-NAQ and ICA-W items to determine whether the two scales measured two conceptually different constructs because, to our knowledge, this study marks the first attempt to measure these two scales simultaneously in the Japanese population. A total of 19 items from the two scales were included in the factor analysis using the maximum likelihood method and Promax rotation. In addition, we performed a Spearman’s rank order correlation test to demonstrate the correlation coefficients between the S-NAQ and ICA-W items.

Following the definition of Leymann [4], when the respondents answered that they had experienced one or more of the items on the S-NAQ and ICA-W at least once a week, they were classified as “TB victim” and “CB victim”, respectively, and coded as 1 for the subsequent analysis. The other cases were classified as “TB non-victim” and “CB non-victim”, and coded as 0. Then, chi-squared and *t*-tests were conducted to compare “TB victim” vs. “TB non-victim” and “CB victim” vs. “CB non-victim”.

Hierarchical binomial logistic regression analyses were performed using TB victimization as an objective variable and the characteristics of the participants as explanatory variables. In all steps, sex and age were forcibly adjusted. In the first step, forward selection (likelihood ratio) was used for the following possible explanatory variables: marital status, annual household income, educational attainment, residential area, managerial position, type of industry, type of job, number of employees in the workplace, average working hours per week, five scales of personality traits, four subscales of the BSJS, two subscales of the OCS-12, and two subscales of the GAWS. In the second step, CB victimization was entered by forward selection in addition to the first step.

Similarly, hierarchical binomial logistic regression analyses were performed using CB victimization as an objective variable; the first model was the same as that described in the previous paragraph, and online information dissemination was added. In the second model, frequency of teleworking was entered in addition to the first model. Lastly, in the third model, TB victimization was entered in addition to the second model. All variables except for sex and age were entered by forward selection.

#### Investigating psychological outcomes for TCB-victimization clusters

We entered 19 variables into the two-step cluster analysis: all nine items of the S-NAQ and all 10 items of the ICA-W. The log-likelihood distance measure was applied for clustering and Akaike’s information criterion (AIC) was used to select the optimal number of clusters. Noise handling was not applied. Then, a series of hierarchical binomial logistic regression analyses were performed to examine the association between the TCB-victimization clusters and mental well-being. The objective variables were psychological distress, insomnia, and loneliness. The clusters gained by the two-step cluster analysis were used as explanatory variables. In the first model, age and sex were controlled. In the second model, forward selection was used to control for the following possible explanatory variables: marital status, annual household income, educational attainment, online information dissemination, residential area, managerial position, type of industry, type of work, number of employees in the workplace, average working hours per week, five scales of personality traits, four subscales of the BSJS, two subscales of the OCS-12, and two subscales of the GAWS.

The two-sided significance level was set at 5%, and SPSS Statistics version 27 (IBM Corp., Armark, NY, USA) was used for all analyses.

## Results

The frequencies of S-NAQ and ICA-W responses are shown in Table 1. The most common S-NAQ response was “Someone withholding information that affects your performance”, with 5.0% of the respondents experiencing this at least once a week. The most common response on the ICA-W was “Your e-mails, phone calls, or messages are ignored at work”, with 5.0% of the respondents experiencing this at least once a week. The factor loading by factor analysis is shown on the right side of Table 1. It was confirmed that each item of the S-NAQ and ICA-W loaded onto each scale separately. Cronbach’s α was 0.94 for the S-NAQ and 0.93 for the ICA-W.

**Table 1:** Frequency of being victimized weekly or daily basis, and the factor loadings of S-NAQ and ICA-W items among regular employees in Japan (*n* = 1,200)

	Being victimized weekly or daily, %	Factor loadings	
		1	2
**S-NAQ items**			
a. Someone withholding information that affects your performance	5.0	**.62**	.00
b. Spreading of gossip and rumors about you	2.7	**.80**	.04
c. Being ignored or excluded	3.4	**.80**	.05
d. Having insulting or offensive remarks made about your person (i.e., habits and background), attitude, or private life	3.0	**.82**	.03
e. Being shouted at or being the target of spontaneous anger (or rage)	3.3	**.82**	-.05
f. Repeated reminders of your errors or mistakes	2.8	**.80**	.01
g. Being ignored or facing a hostile reaction when you approach	3.3	**.84**	.05
h. Persistent criticism of your work and effort	3.8	**.86**	-.06
i. Practical jokes carried out by people you do not get along with	2.2	**.81**	.03
**ICA-W items**			
A. Your e-mails, phone calls, or messages are ignored at work	5.0	.10	**.43**
B. Your e-mails are forwarded to third parties in order to harm you	1.6	.05	**.81**
C. Your work is criticized publicly by means of ICT	1.5	-.01	**.89**
D. Somebody is withholding e-mails or files you need, making your work more difficult	2.9	.08	**.61**
E. Rumors or gossip is being spread about you by means of ICT	1.2	-.01	**.93**
F. You are being insulted, threatened, or intimidated by means of ICT	1.4	.03	**.88**
G. Constant remarks are being made about you and your private life by means of ICT	1.8	.01	**.89**
H. Your personal information is hacked and used to harm you	1.3	-.02	**.87**
I. Somebody shares photos or videos of you on the Internet to make fun of you	1.2	-.04	**.87**
J. Somebody takes over your identity	1.1	-.06	**.86**

^a^*S-NAQ* Short Negative Act Questionnaire

^b^*ICA-W* Inventory of Cyberbullying Acts at Work

The Spearman’s rank correlation coefficients of S-NAQ and ICA-W items are shown in Table 2. The correlation coefficients were ranged from 0.211 to 0.467. Relatively strong correlation coefficients were found between “c. Being ignored or excluded” and “B. Your e-mails are forwarded to third parties in order to harm you” (0.465), and between “i. Practical jokes carried out by people you do not get along with” and “F. You are being insulted, threatened, or intimidated by means of ICT” (0.467).

**Table 2:** Spearman’s rank correlation coefficients^a^ between S-NAQ^b^ and ICA-W^c^ items (*n* = 1,200)

	ICA-W items									
	A	B	C	D	E	F	G	H	I	J
S-NAQ Items										
a	.305	.295	.290	.309	.250	.300	.289	.237	.250	.211
b	.269	.400	.338	.334	.362	.422	.393	.359	.342	.341
c	.311	.465	.382	.341	.394	.441	.420	.387	.359	.377
d	.270	.389	.364	.290	.378	.423	.405	.361	.340	.348
e	.274	.358	.326	.283	.326	.393	.356	.323	.307	.313
f	.255	.353	.354	.284	.317	.364	.349	.312	.315	.296
g	.305	.451	.394	.329	.401	.448	.407	.394	.352	.364
h	.294	.361	.335	.300	.322	.373	.354	.320	.307	.307
i	.305	.452	.403	.348	.436	.467	.432	.426	.377	.370

^a^For all pairs, the *p*-values were < .001

^b^*S-NAQ* Short Negative Act Questionnaire

^c^*ICA-W* Inventory of Cyberbullying Acts at Work

See Table 1 for the descriptions of each S-NAQ and ICA-W item

The characteristics of the participants and the percentages of TB/CB victimization are shown in Table 3. The results of the chi-squared test regarding the association between the percentages of TB/CB victimization and characteristics of the participants are listed together. The percentages of participants who had experienced TB and CB victimization were 11.3% and 8.0%, respectively. Sex and managerial position were significantly associated with TB victimization. The percentages of TB victimization were significantly higher for males than for females and for managers than for non-managers. Age, educational attainment, position, average working hours per week, and frequency of teleworking were significantly associated with CB victimization. The percentages of CB victimization were significantly higher for managers than for non-managers. No statistical significance was found in regarding to residential area, type of industry, type of work, or number of employees in the workplace.

**Table 3:** Characteristics of the participants and percentage of TB^a^/CB^b^ victimization (*n* = 1,200)

	Number	TB victim, %	*P*	CB victim, %	*P*
**Overall**	1,200	11.3		8.0	
**TB victim**					
No	1,064			5.4	< .001
Yes	136			28.7	
**CB victim**					
**No**	1,104	8.8	< .001		
Yes	96	40.6			
**Sex**					
Male	800	12.6	.046	8.9	.11
Female	400	8.8		6.3	
**Marital status**					
Not married	497	10.3	.33	7.2	.42
Married	703	12.1		8.5	
**Annual household income, JPY**					
4 million or less	264	11.4	.76	6.4	.19
4–8 million	591	11.3		7.4	
8–12 million	249	12.4		11.2	
12 million or more	96	8.3		7.3	
**Educational attainment**					
High school	250	9.2	.48	2.8	.001
College, etc	157	11.5		5.7	
University/graduate school	793	12.0		10.1	
**Active dissemination via SNSs, blog, or video-sharing site**					
No	1,057	10.4	.006	6.1	< .001
Yes	143	18.2		21.7	
**Residential area**					
Hokkaido/Tohoku	83	15.7	.24	6.0	.18
Tokyo	254	15.4		10.6	
Kanto (excluding Tokyo)	370	9.5		6.2	
Chubu	146	9.6		6.2	
Kansai	207	10.1		11.1	
Chugoku/Shikoku	73	11.0		8.2	
Kyusyu/Okinawa	67	9.0		4.5	
**Type of industry**					
Construction	62	6.5	.33	11.3	.53
Manufacturing	333	11.4		6.9	
Information/communication	107	11.2		11.2	
Transportation	66	7.6		10.6	
Wholesale/retail trade	96	17.7		7.3	
Finance/insurance/real estate	110	12.7		9.1	
Healthcare/welfare	90	13.3		3.3	
Services	132	9.1		7.6	
Public sector	64	10.9		10.9	
Academic research	40	2.5		2.5	
Others	100	14.0		9.0	
**Type of work**					
Professional/technical position	296	10.1	.30	10.1	.26
Clerical position	489	10.6		8.6	
Sales position	77	18.2		5.2	
Service position	98	15.3		4.1	
Production engineering	91	9.9		4.4	
Others	149	10.7		8.1	
**Position**					
Non-manager	946	10.4	.04	6.6	< .001
Manager	254	15.0		13.4	
**Number of employees in the workplace**					
5 or fewer	175	11.4	.71	4.6	.33
6–9	161	11.2		8.1	
10–19	242	9.1		7.0	
20–29	124	10.5		9.7	
30 or more	498	12.7		9.2	
**Average working hours per week**					
30 or fewer	59	16.9	.09	15.3	.04
30–39	252	10.7		7.1	
40–49	649	9.6		6.5	
50–59	142	14.8		12.0	
60 or more	98	16.3		10.2	
**Frequency of teleworking**					
Almost never	777	11.1	.25	4.5	< .001
About 1 to 3 times a month	79	17.7		19.0	
About 1 to 3 times a week	203	9.4		15.3	
Almost every day	141	12.1		10.6	

^a^*TB* Traditional bullying defined by Short Negative Act Questionnaire

^b^*CB* Cyberbullying defined by Inventory of Cyberbullying Acts at Work

The mean scores for each scale regarding TB/CB victimization are shown in Table 4. The results of a *t*-test for the difference in mean scores between TB/CB victim and non-victim are listed together. Regarding job stress, TB/CB victims had significantly higher mean scores for stress-enhancing factors than did non-victims. Regarding personality traits, TB/CB victims had significantly lower mean scores for agreeableness than did non-victims. Regarding psychological distress, TB/CB victims had significantly higher mean K6 scores than did non-victims. Regarding loneliness, TB/CB victims had significantly higher mean TIL-J scores than did non-victims. Regarding organizational climate, TB/CB victims had significantly higher mean scores on the tradition scale than did non-victims.

**Table 4:** Mean scores of each scale for TB^a^/CB^b^ victimization (*n* = 1,200)

	Range	TB victim			CB victim		
		No	Yes	*P*	No	Yes	*P*
**Age**	21–64	40.64	39.57	.26	40.90	36.00	< .001
**Japanese version of the Ten-Item Personality Inventory**							
Extraversion	2–14	7.17	7.63	.02	7.19	7.53	.13
Agreeableness	2–14	9.47	8.38	< .001	9.39	8.77	.007
Conscientiousness	2–14	7.84	7.74	.66	7.80	8.08	.26
Neuroticism	2–14	8.03	8.60	.008	8.11	8.01	.71
Openness	2–14	7.30	7.66	.09	7.28	8.06	< .001
**Brief Scales for Job Stress**							
Quantitative workload	1–4	2.14	2.43	< .001	2.14	2.49	< .001
Qualitative workload	1–4	2.11	2.47	< .001	2.11	2.61	< .001
Job control	1–4	2.51	2.35	.02	2.49	2.57	.31
Support from colleagues and superiors	1–4	2.46	2.24	.001	2.43	2.48	.42
**the 12-item Organizational Climate Scale**							
Tradition scale	1–2	1.31	1.52	< .001	1.33	1.44	.001
Organizational environment scale	1–2	1.35	1.32	.32	1.35	1.36	.73
**Gratitude at Work Scale**							
Gratitude for supportive work environment	1–5	3.07	2.71	< .001	3.04	2.99	.63
Gratitude for meaningful work	1–5	3.12	2.96	0.052	3.09	3.18	.36
**the 6-item Kessler Psychological Distress Scale**	0–24	4.91	9.58	< .001	5.19	8.23	< .001
**Athens Insomnia Scale**	0–24	5.82	9.77	< .001	6.09	8.29	< .001
**Japanese version of the Three-Item Loneliness scale**	3–9	4.70	6.18	< .001	4.81	5.51	< .001

^a^*TB* Traditional bullying defined by Short Negative Act Questionnaire

^b^*CB* Cyberbullying defined by Inventory of Cyberbullying Acts at Work

The results of hierarchical binomial logistic regression analysis using TB victimization as the dependent variable are shown in Table 5. Extraversion, agreeableness, qualitative workload, and support from colleagues and superiors were entered into step 1 by forward selection. In step 2, CB victimization was added. All explanatory factors except qualitative workload maintained statistical significance.

**Table 5:** Factors correlated with TB^a^ victimization (*n* = 1,200)

	Step 1		Step 2	
	OR	(95% CI)	OR	(95% CI)
Female (ref. Male)	0.73	(0.48–1.14)	0.76	(0.48–1.18)
Age	0.99	(0.97–1.01)	1.00	(0.98–1.02)
Extraversion	1.15	(1.06–1.25)	1.14	(1.04–1.24)
Agreeableness	0.83	(0.76–0.91)	0.84	(0.77–0.92)
Qualitative workload	1.42	(1.10–1.83)	1.29	(0.99–1.68)
Support from colleagues and superiors	0.67	(0.50–0.89)	0.62	(0.45–0.83)
Tradition scale	1.32	(1.20–1.47)	1.30	(1.17–1.45)
CB^b^ victim, Yes (ref. No)			5.61	(3.37–9.33)
Nagelkerke *R*^2^	0.16		0.22	

^a^*TB* Traditional bullying defined by Short Negative Act Questionnaire

^b^*CB* Cyberbullying defined by Inventory of Cyberbullying Acts at Work

Statistical analyses were conducted using hierarchical binomial logistic regression with forward selection (likelihood ratio)

The results of hierarchical binomial logistic regression analysis using CB victimization as the dependent variable are shown in Table 6. Managerial position, active dissemination via SNSs, etc., openness, and qualitative workload were entered into step 1 by forward selection. Frequency of teleworking and TB victimization were selected to be added in steps 2 and 3, respectively. In the final step, the correlation between openness CB victimization was attenuated by adding other explanatory variables. On the other hand, managerial position (odds ratio [OR] = 1.90, 95% confidence interval [CI] = 1.09–3.30), active dissemination by SNSs, etc. (OR = 2.59, 95% CI = 1.42–4.30), qualitative workload (OR = 1.84, 95% CI = 1.34–2.52), and frequency of teleworking (“almost never” vs. “about 1 to 3 times a month”: OR = 3.09, 95% CI = 1.44–6.62; “about 1 to 3 times a week”, OR = 3.46, 95% CI = 1.96–6.11) maintained statistical significance. Regarding the frequency of teleworking, it is noteworthy that the difference between “almost never” and “almost every day” was not statistically significant.

**Table 6:** Factors correlated with CB^a^ victimization (*n* = 1,200)

	Step 1		Step 2		Step 3	
	OR	(95% CI)	OR	(95% CI)	OR	(95% CI)
Female (ref. Male)	0.87	(0.53–1.45)	0.89	(0.53–1.49)	0.89	(0.52–1.53)
Age	0.95	(0.92–0.97)	0.95	(0.93–0.98)	0.95	(0.93–0.98)
Manager (ref. Non-manager)	2.35	(1.40–3.95)	1.97	(1.16–3.36)	1.90	(1.09–3.30)
Active dissemination via SNSs, blog, or video-sharing site, Yes (ref. No)	2.90	(1.74–4.83)	2.74	(1.63–4.63)	2.59	(1.42–4.30)
Openness	1.11	(1.001–1.23)	1.10	(0.995–1.23)	1.08	(0.97–1.20)
Qualitative workload	2.06	(1.55–2.73)	2.03	(1.51–2.73)	1.84	(1.34–2.52)
Frequency of teleworking (ref. Almost never)						
About 1 to 3 times a month			3.44	(1.70–6.99)	3.09	(1.44–6.62)
About 1 to 3 times a week			2.94	(1.71–5.06)	3.46	(1.96–6.11)
Almost every day			1.95	(1.001–3.79)	1.96	(0.97–3.95)
TB^b^ victim, Yes (ref. No)					6.03	(3.60–10.10)
Nagelkerke *R*^2^	0.17		0.21		0.29	

^a^*CB* Cyberbullying defined by Inventory of Cyberbullying Acts at Work

^b^*TB* Traditional bullying defined by Short Negative Act Questionnaire

Statistical analyses were conducted using hierarchical binomial logistic regression with forward selection (likelihood ratio)

The two-step cluster analysis revealed three TCB-victimization clusters. The AIC was 8,502.32 when the number of clusters was two, 6,560.20 when the number of clusters was three, and 6,082.60 when the number of clusters was four. The ratio of the AIC change was 0.264 between two and three clusters and dropped to 0.065 between three and four clusters. A total of 81.0% (*n* = 972) of the respondents were assigned to cluster X, 14.3% (*n* = 171) to cluster Y, and 4.8% (*n* = 57) to cluster Z. The ratio of the biggest/smallest cluster size was 17.1. According to a precedent study [72], the overall model quality was “good”, with an average silhouette of 0.7. Based on the between-cluster comparisons, as shown in Table 7, the respondents in cluster X scored almost 1 for all 19 items, which means they rarely experienced negative acts as illustrated by the S-NAQ and ICA-W. The respondents in cluster Y showed a different pattern compared with those in cluster X, with scores for S-NAQ items ranging from 1.88 to 2.48. The scores for ICA-W items were higher, but not that significant (1.01–1.67). Lastly, the respondents in cluster Z showed a distinct pattern among the three clusters, demonstrating the highest score for all 19 items of the S-NAQ (2.60–2.88) and ICA-W (2.53–3.05). In total, the respondents in cluster X were nearly free from both TB and CB victimization. The respondents in cluster Y experienced TB victimization more frequently, but that was not the case for CB victimization. The respondents in cluster Z were characterized as frequent targets of both TB and CB.

**Table 7:** Mean scores for each S-NAQ^a^ and ICA-W^b^ item by three clusters (*n* = 1,200)

	Cluster X (*n* = 972)	Cluster Y (*n* = 171)	Cluster Z (*n* = 57)	Statistical significance by post-hoc Dunn–Bonferroni test
**S-NAQ items**				
a	1.28	2.48	2.72	X < Y, Z
b	1.06	2.23	2.81	X < Y < Z
c	1.03	2.16	2.88	X < Y < Z
d	1.04	2.12	2.77	X < Y < Z
e	1.10	2.33	2.60	X < Y, Z
f	1.09	2.23	2.72	X < Y, Z
g	1.02	2.15	2.91	X < Y < Z
h	1.06	2.27	2.65	X < Y, Z
i	1.02	1.88	2.60	X < Y < Z
**ICA-W items**				
A	1.19	1.67	2.91	X < Y < Z
B	1.01	1.19	2.95	X < Y < Z
C	1.02	1.04	3.05	X, Y < Z
D	1.07	1.36	3.02	X < Y < Z
E	1.01	1.02	2.82	X, Y < Z
F	1.00	1.06	3.11	X < Y < Z
G	1.01	1.05	3.02	X, Y < Z
H	1.00	1.03	2.67	X, Y < Z
I	1.01	1.01	2.53	X, Y < Z
J	1.01	1.06	2.53	X, Y < Z

^a^*S-NAQ* Short Negative Act Questionnaire

^b^*ICA-W* Inventory of Cyberbullying Acts at Work

See Table 1 for the descriptions of each S-NAQ and ICA-W item

To examine the potential differences among TCB-victimization clusters regarding the association with psychological well-being, we performed a series of hierarchical binomial logistic regression analyses. The results are shown in Table 8, 9 and 10. In model 1, with sex and age as the explanatory variables, clusters Y and Z had significantly higher ORs for psychological distress, insomnia, and loneliness, with cluster X as the reference. In model 2, after adjusting for possible confounders, clusters Y and Z maintained their statistical significance. As for cluster Y, the ORs for psychological distress, insomnia, and loneliness were 3.70 (95% CI = 2.37–5.80), 3.33 (95% CI = 2.18–5.07), and 2.83 (95% CI = 1.92–4.19), respectively. As for cluster Z, the ORs for psychological distress, insomnia, and loneliness were 12.63 (95% CI = 4.20–38.03), 6.26 (95% CI = 2.80–14.01), and 3.24 (95% CI = 1.74–6.04), respectively.

**Table 8:** Association between TCB-victimization^a^ clusters and psychological distress^b^ (*n* = 1,200)

	Step 1		Step 2	
	OR	(95% CI)	OR	(95% CI)
Female (ref. male)	1.10	(0.85–1.42)	1.55	(1.14–2.10)
Age	0.98	(0.97–0.99)	0.99	(0.97–0.999)
Cluster Y (ref. cluster X)	6.46	(4.28–9.75)	3.70	(2.37–5.80)
Cluster Z (ref. cluster X)	16.92	(6.06–47.29)	12.63	(4.20–38.03)
Active dissemination via SNSs, blog, or video sharing site, Yes (ref. No)			1.85	(1.16–2.93)
Extraversion			0.92	(0.87–0.98)
Agreeableness			0.89	(0.82–0.95)
Neuroticism			1.23	(1.15–1.31)
Qualitative workload			2.13	(1.76–2.59)
Support from colleagues and superiors			0.63	(0.51–0.79)
Tradition scale			1.11	(1.03–1.21)
Gratitude for meaningful work			0.76	(0.64–0.91)
Nagelkerke *R*^2^	0.18		0.40	

^a^*TCB* Traditional and cyber bullying

^b^Defined by the 6-item Kessler Psychological Distress Scale, with total score 5 or more

Statistical analyses were conducted using hierarchical binomial logistic regression with forward selection (likelihood ratio)

**Table 9:** Association between TCB-victimization^a^ clusters and insomnia^b^ (*n* = 1,200)

	Step 1		Step 2	
	OR	(95% CI)	OR	(95% CI)
Female (ref. male)	0.92	(0.71–1.18)	1.09	(0.82–1.44)
Age	0.99	(0.98–1.00)	1.00	(0.98–1.01)
Cluster Y (ref. cluster X)	4.92	(3.30–7.35)	3.33	(2.18–5.07)
Cluster Z (ref. cluster X)	6.81	(3.18–14.58)	6.26	(2.80–14.01)
Not married (ref. married)			0.73	(0.55–0.96)
Manager (ref. non-manager)			1.49	(1.06–2.10)
Neuroticism			1.19	(1.12–1.26)
Quantitative workload			1.31	(1.05–1.63)
Qualitative workload			1.48	(1.18–1.87)
Job control			0.81	(0.68–0.97)
Organizational environment scale			0.91	(0.84–0.98)
Gratitude for supportive work environment			0.72	(0.60–0.86)
Nagelkerke *R*^2^	0.12		0.27	

^a^*TCB* Traditional and cyber bullying

^b^Defined by Athens Insomnia Scale, with total score 6 or more

Statistical analyses were conducted using hierarchical binomial logistic regression with forward selection (likelihood ratio)

**Table 10:** Association between TCB-victimization^a^ clusters and loneliness^b^ (*n* = 1,200)

	Step 1		Step 2	
	OR	(95% CI)	OR	(95% CI)
Female (ref. male)	1.00	(0.77–1.29)	1.11	(0.81–1.51)
Age	0.99	(0.98–1.00)	1.00	(0.98–1.01)
Cluster Y (ref. cluster X)	4.07	(2.88–5.74)	2.83	(1.92–4.19)
Cluster Z (ref. cluster X)	3.00	(1.73–5.20)	3.24	(1.74–6.04)
High school (ref. university/graduate school)			0.73	(0.50–1.05)
College, etc. (ref. university/graduate school)			1.47	(0.97–2.23)
4 million or less (ref. 4–8 million)			1.63	(1.15–2.32)
8–12 million (ref. 4–8 million)			1.04	(0.73–1.50)
12 million or more (ref. 4–8 million)			0.97	(0.55–1.71)
Extraversion			0.84	(0.79–0.89)
Agreeableness			0.86	(0.81–0.93)
Neuroticism			1.16	(1.08–1.24)
Qualitative workload			1.36	(1.12–1.65)
Job control			0.54	(0.43–0.68)
Tradition scale			1.18	(1.09–1.28)
Organizational environment scale			0.86	(0.79–0.93)
Nagelkerke *R*^2^	0.09		0.34	

^a^*TCB* Traditional and cyber bullying

^b^Defined by Japanese version of the Three-Item Loneliness scale with total score 6 or more

Statistical analyses were conducted using hierarchical binomial logistic regression with forward selection (likelihood ratio)

## Discussion

### Prevalence of TB/CB victimization among regular employees in Japan

To our knowledge, this study was the first attempt to investigate the prevalence of CB among employees in Japan using a rating scale that can be compared with precedent studies. The results indicated that 8.0% of the respondents had experienced some form of CB at least once a week. Precedent research in Europe has pointed out that the prevalence rate of CB victimization varies from 9 to 21% [18]; the result of the present study was below that range. A study in Korea [29] showed that 8.0% of participants experienced at least one incident of CB from peers or superiors every week or every day for the past 6 months, which was similar to our results. Regional and cultural differences between Europe and Asia might have affected the differences in CB victimization rates. However, research on CB is concentrated on the youth in Asian countries; little research has been conducted on CB at work. Further studies are needed to clarify the background to the prevalence of CB in the workplace. In addition, this study had a cross-sectional design, so our results are only for a single point in time when teleworking was rapidly introduced. It would be interesting to conduct a fixed-point survey in the future to observe trends in the incidence of CB.

In addition, this study used an established rating scale to examine the prevalence of TB among employees. We found that 11.3% of the respondents had experienced some form of TB at least once a week. In a meta-analysis of precedent studies, the percentage of employees who were bullied at least once a week was 14.6% [24]. The results of precedent studies in Japan have reported that the percentage of those who were bullied once a week or more ranged from 9.0% [52] to 9.7% [73] which was lower compared to our results. The most frequently experienced TB event was “someone withholding information that affects your performance”, which was also in accordance with a precedent study [52, 73].

### Explanatory factors related to workplace TB/CB victimization

It is interesting to note that a strong association was found between managerial position and CB victimization, which is consistent with precedent studies [11, 18]. In precedent studies, this tendency has been explained as follows: those who are in a weak position in a workplace, such as non-managers, might take advantage of the anonymity on the Internet to exact revenge on those who are in a stronger position, such as managers [74]. It has also been pointed out that managers use ICT devices more frequently in their daily work than do non-managers, which might help explain the association between managerial position and CB victimization [18]. Although this survey did not investigate whether the respondents is a middle manager, it is possible that their position is a factor that makes them vulnerable to bullying victimization. Middle managers are subjected to pressure from higher-level managers on behalf of their departments. They are also taking responsibility for the actions of their subordinates. In Japan, the term “playing manager” is often used to describe the fact that managers are responsible not only for the administrative aspects of their work, but also for the practical aspects.

Our results revealed that active dissemination via SNSs was significantly associated with CB victimization, in line with precedent studies [75, 76]. A precedent study targeting undergraduates in the US and Australia reported that posting negative content and having Facebook friends who posted such content were both strong predictors of CB victimization [34]. Although we did not assess the content of the postings, if done anonymously or using real names, or if work-related or for private use, our results showed that active information dissemination was also a predictor of CB victimization among employees in Japan. Online information dissemination is becoming increasingly popular, especially among the younger generations. Therefore, attention should be paid to the potential risk of becoming a victim of CB when using social media. Further research is warranted on ways to avoid CB while enjoying online activities, or on how to identify those who fall victim to CB quickly.

The results of the hierarchical logistic regression analysis revealed that high openness was associated with CB in the first model. However, after adjusting for the frequency of teleworking, this association was no longer significant. The results of precedent studies regarding the association between personality traits and CB victimization have been inconsistent. One study found that openness and extroversion were associated with CB victimization among young adults [34], while another reported that the probability of CB victimization increased with increasing levels of extraversion, neuroticism, openness, and agreeableness [77]. It is possible that personality traits such as high openness are not direct factors for CB victimization, but rather, indirect factors via behaviors or conditions such as teleworking. Since there has been a lack of consistent results regarding the relationship between CB in the workplace and employees’ personality traits [11], more knowledge needs to be accumulated.

In line with a precedent study [78], our results revealed that scoring lower on agreeableness and higher on extraversion were significantly associated with TB victimization. Whether the relationship between TB victimization and personality traits is an antecedent or a consequence remains a topic of debate [79]. From the standpoint of antecedents, those scoring lower on agreeableness and higher on extraversion have poorer social skills and are more likely to experience TB victimization [80]. However, from the standpoint of consequences, experiencing negative treatment such as TB victimization decreases one’s tendency toward cooperation and lowers agreeableness [33]. Precedent studies have argued that bullying is a prolonged stress that may significantly impact personality. It has also been reported that personality traits can change throughout life, such as decreasing neuroticism scores throughout adulthood [81]. Therefore, training programs that improve social skills and change individual personality traits might help reduce the negative effects of TB victimization.

In the present study, we also showed that TB victimization was significantly associated with a lower level of support from colleagues and superiors, whereas CB victimization was significantly associated with a higher level of qualitative workload. Our results also revealed that scoring higher on the tradition scale, which indicates a coercive, commanding, and legalistic organizational climate, was significantly associated with victimization. Those results were consistent with precedent findings that working characteristics such as disruptive leadership, a lack of autonomy at work, team conflict, ineffective organizational strategies, role ambiguity, a competitive atmosphere, high workloads, excessive pressure, and a lack of social support were associated with a higher incidence of TB [15, 82–85]. It has also been reported that bullying occurs more frequently in workplaces with a “climate of bullying” [46, 86, 87]. In such organizations, it might be difficult for employees to cooperate with each other, and it is more likely that employees will take advantage of or interfere with others; such organizations can also induce negative emotions such as anger, fear, and sadness [88]. Although the present study depends on employees’ perceptions of their workplace, not on the observational state, managers should take care to not allow the company to fall into an organizational climate where bullying is tolerated.

Regarding the relationship between CB victimization and teleworking, to our knowledge, this is the first study in Japan to report a significant association between teleworking and the incidence of CB victimization, after controlling for other possible explanatory factors. Therefore, our results partially support hypothesis 2. Precedent studies have pointed out that shifting to remote work leads to a reduction in the social support of the workplace and that a continuous online environment can cause exhaustion, which could lead to CB [13, 89]. Although teleworking is expected to continue even after the COVID-19 pandemic, the possibility that teleworkers may be more susceptible to CB victimization should be considered. It is also worth noting that the ORs were higher for those who teleworked 1–3 times a week than for those who teleworked exclusively. Frequent switching of working locations might be a factor in the occurrence of CB. However, in this study, we measured only the frequency of teleworking; we did not collect information on where or how long individual workers were teleworking. Further research is needed to assess the telework environment in greater depth and disentangle the relationships between CB and teleworking.

### Relationships between psychological well-being and workplace TCB-victimization clusters

In the present study, we found significant relationships between TCB-victimization clusters and employee well-being; that is, psychological distress, insomnia, and loneliness. For all three types of negative outcomes, belonging to the cluster Z showed higher ORs than belonging to the cluster Y. Thus, our third hypothesis was confirmed. Our study is noteworthy in that it considered the combination of TB and CB victimization and identified associations with psychological distress, insomnia, and loneliness, thereby providing new evidence to that from precedent studies in which TB and CB were examined separately [51]. Our results underscore the important point that attention should be paid to not only TB, but also the coexistence of CB. Our findings regarding the relationship between TB at work and depression [51], insomnia [3, 90], and social isolation [91, 92] are consistent with those from precedent research. It has been reported that CB victimization among adolescents is associated with loneliness [93, 94] and sleep problems [95]. Our study expands the existing literature showing the negative effects of CB victimization on sleep and loneliness among employees. Some researchers have argued that CB victimization is merely an extension of TB victimization, and as such, the impact should not be overestimated [96]. However, in the present study, the combination of TB and CB victimization was found to have a stronger negative impact on mental health, suggesting the importance of countermeasures against both CB and TB victimization.

Gratitude for work appeared to predict well-being: a higher level of gratitude was associated with a lower risk of psychological distress and sleep problems, which is in line with precedent studies [97–99]. Researchers are currently developing various intervention programs to increase gratitude in the workplace [100, 101], which could be a promising way to suppress the negative effects of CB and TB victimization on well-being. By contrast, gratitude at work was not found to predict TB or CB victimization. A precedent study involving adolescents reported that gratitude promoted prosocial behaviors among bystanders toward bullying victims, resulting in a reduction in bullying [102]. Although the role of bystanders is beyond the scope of the present study, it would be interesting to examine how the workplace positive interaction works to prevent TB/CB in a future study.

### Implications for practice and future research

The results of the present study revealed that being a manager, having a high qualitative workload, teleworking about once a month to three times a week, actively disseminating information on the Internet, and TB victimization were associated with CB victimization. We also found that the coexistence of TB/CB victimization caused depression, insomnia, and loneliness. These findings have important practical implications because they clarify the characteristics of employees in Japan who are prone to CB victimization and its negative consequences for the first time. It has been pointed out that employees are more susceptible to CB than to TB victimization outside the workplace and during nonworking hours, and that they tend to keep their problems to themselves and feel isolated because they are less visible to others [35]. Japanese employees tend to be trained to manage their negative emotions on their own to maintain rapport with others, in contrast to individualistic-oriented cultures [103]. Because of this tendency, victims might not be able to confide in coworkers about their bullying and may fall into a negative spiral of distress, resulting in job turnover. Therefore, it is necessary not only for each worker to take care not to be involved in CB, but also for companies to take organizational countermeasures to prevent the occurrence and promote the early detection of CB.

### Limitations

This study also has some limitations. Caution is needed when generalizing our results to other populations because the participants were registered monitors of an Internet research company, which suggests a possible sampling bias. Because such individuals are likely to be more familiar with the Internet, the prevalence of CB could have been overestimated. Additionally, since the percentage of TB victimization in the current study was higher than in previous studies in Japan, it is possible that the participants were more likely to report bullying-related behaviors. Therefore, the percentage of CB victimization might be overestimated. On the other hand, if those who had workplace victimization have already left their jobs, this study would underestimate the percentage of bullying victimization. More representative sample should be obtained in future studies. Only regular employees who were not on leave were included in the analysis; non-regular employees and those who were currently on leave were not included. Such populations should be considered in future studies, as diversity in work styles becomes more common. In addition, the validity and reliability of the Japanese versions of the S-NAQ and ICA-W have not been confirmed, and no information is available on the severity of bullying, perpetrators, or bystanders. This study did not identify the perpetrator, which should be taken into consideration for further research. Not all types of workplace bullying are not considered: e.g. sexual harassment could not be identified by the S-NAQ or ICA-W. This study did not consider all the other factors that have been pointed out in precedent studies to affect employees’ well-being. Other factors that influence employees’ well-being include, for example, life events in the family and events in childhood [104]; these need to be considered in a future study. Moreover, the duration of teleworking experience was not elicited, so the effect of employees becoming accustomed to teleworking was not considered. Lastly, this was a cross-sectional study, so no causal relationships could be determined. For example, employees in a state of high psychological distress are more likely to perceive work-related contacts as a negative event [105]. A longitudinal study is needed to verify the causal relationships of workplace bullying and negative health consequences.

## Conclusions

To our knowledge, this is the first epidemiological survey on TB/CB victimization using internationally comparable scales among employees in Japan. The results revealed the prevalence of TB/CB victimization, the characteristics of TB/CB victimization, and the relationship between TCB-victimization clusters and psychological well-being. These findings could be the first step in Japan to develop effective countermeasures to prevent the incidence and adverse outcomes of TB and CB.

## Supplementary Information

**Additional file 1.** Categorization of the variables.

## Data Availability

The datasets used and analysed during the current study are available from the corresponding author on reasonable request.

## Abbreviations

AIC: Akaike’s information criterion
AIS: Athens Insomnia Scale
BSJS: Brief Scales for Job Stress
CB: Cyberbullying
CI: Confidence interval
COVID-19: Coronavirus disease 2019
FY: Fiscal year
GAWS: Gratitude at Work Scale
ICA-W: Inventory of Cyberbullying Acts at Work
ICT: Information and communication technology
JDCS: Job Demand–Control–Support
K6: The 6-item Kessler Psychological Distress Scale
NAQ: Negative Acts Questionnaire
OCS-12: The 12-item Organizational Climate Scale
OR: Odds ratio
S-NAQ: Short form of NAQ
TB: Traditional bullying
TCB: Traditional and cyber bullying
TIL-J: Japanese version of the Three-Item Loneliness Scale
TIPI-J: Japanese version of the Ten-Item Personality Inventory
